# Eco-dyeing with biocolourant based on natural compounds

**DOI:** 10.1098/rsos.171134

**Published:** 2018-01-24

**Authors:** Fubang Wang, Jixian Gong, Yanfei Ren, Jianfei Zhang

**Affiliations:** 1 School of Textiles, Tianjin Polytechnic University, Tianjin 300387, People's Republic of China; 2 Key Laboratory for Advanced Textile Composites of the Education Ministry of China, Tianjin 300387, People's Republic of China

**Keywords:** biomass pigments, tea polyphenols, dyeing, residual liquor, *Integ* values

## Abstract

Biomass pigments have been regarded as promising alternatives to conventional synthetic dyestuffs for the development of sustainable and clean dyeing. This investigation focused on *in situ* dyeing of fabrics with biopigments derived from tea polyphenols via non-enzymatic browning reaction. The average particle size of dyed residual liquor with natural tea polyphenol was 717.0 nm (ranging from 615.5 to 811.2 nm), and the *Integ* value of dyed wool fabrics was the greatest compared to those of counterparts. In addition, the *Integ* values of dyed fabrics with residual liquor were much bigger than those with the first reaction solutions when dyed by identical dyeing liquor. As a result, the dyeing process could be carried out many times because the concentration of the residual liquor was relatively superior. All dyed fabrics acquired admirable rubbing as well as washing fastness, and the relevant dyeing mechanism has been analysed in the paper.

## 1. Introduction

As green and sustainable development has become the needed trend, a constantly increasing interest in biomass pigments has been aroused in the textile industry in recent years [1,2]. That is regarded as an ecological and sustainable dyeing technology to address environmental contamination issues caused by the application of synthetic dyestuffs [3–5].

Biomass pigments are better than synthetic dyestuffs that are applied currently [6], especially with regard to aspects concerning sustainability of the source, environmental conservation during manufacture and the ecology of dyed textiles [7,8]. Biomass pigments have broad application prospects if they are applied to underclothes, and provide new opportunities for the production of high-end ecological textiles [9,10]. Biomass pigments are synthesized from organisms [11,12], and there is a promising perspective for the application of biomass dyes to address environmental contamination issues caused by synthetic dyestuffs [13,14].

**Figure 1.**
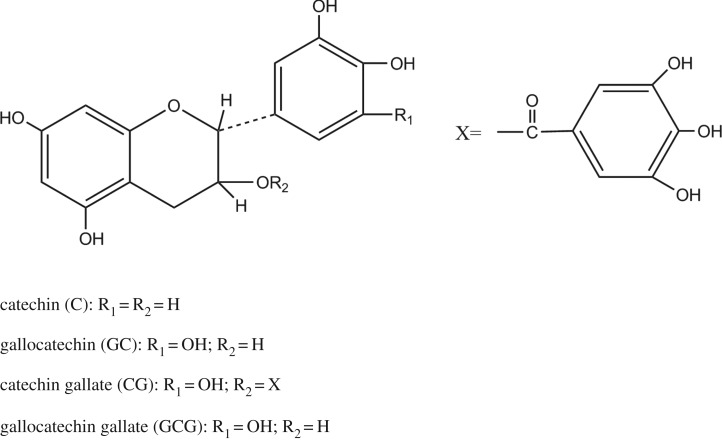
Chemical structural formula of tea polyphenols.

Tea polyphenol (TP) is the main component of green tea, which could be determined to be catechin (C), gallocatechin (GC), catechin gallate (CG) and gallocatechin gallate (GCG) (figure 1) [15,16]. Tea is a renewable biomass resource that is grown in many nations and regions around the world. In addition, relevant investigation demonstrates that tea polyphenols possess diverse healthy functions, such as anticancer, antidiabetic as well as antimicrobial.

Tea polyphenol is the precursor of tea pigments, which is a sort of natural functional compound extracted from green tea and could be transformed into tea pigments via oxidative polymerization [17]. Tea polyphenol could be oxidized to quinone intermediates under high temperature or alkaline conditions, which would be transformed into theaflavin promptly on the grounds that quinone intermediates are unstable [18], and theabrownin would be generated via a non-enzymatic browning reaction between theaflavin and amino acid. Addition of exogenous additives not only could enhance the content of tea pigments, but also endow a pleasant aroma to dyed fabrics, which could achieve the processing simultaneously by both dyeing and finishing accordingly [19].

Amino acid is the general term of organic compounds that contain amino groups as well as carboxy groups and widely exist in the natural world, which are also the basic component unit of biological functional protein. Amino acids are able to form in plant or animal tissue and also could be obtained through proteolysis, which is one of the renewable biomass resources of living organisms, and is extensively applied in many industries. Amino acid is related to the formation of tea pigments, being involved in the process of conversion between tea polyphenols and tea pigments.

In this work, the dyeing process via reaction between tea polyphenols and glycine was studied, and the colour fastness of the dyed fabrics was measured. In addition, the possible reaction mechanisms between tea polyphenols and glycine as well as the dyeing mechanism were analysed.

## 2. Material and methods

### 2.1. Materials

#### 2.1.1. Plant materials

Food grade tea polyphenol was purchased from Liyuan food additives limited company of Guangzhou in Guangdong Province of China, which was obtained via decolourization processing, and the conent of the effective substance was 99%. Natural tea polyphenol was orange in colour with an effective substance content of 98%; this was from green tea extract and purchased from True Taste Food limited company in Shanghai city in China.

#### 2.1.2. Textile materials

The wool fabric (warp density 86 yarns, weft density 51 yarns per inch; weight 132.0 g m^−2^; thickness 0.45 mm) was purchased from Jiangsu Huaxi Spinning limited company in East China. The silk fabric (warp density 325 yarns per inch, weft density 34 yarns per inch; weight 75.0 g m^−2^) was bought from Fing Silk limited company of Hangzhou, the capital of Zhejiang Province in South China. The cotton fabric (warp density 77 yarns per inch, weft density 66 yarns per inch; weight 168 g m^−2^), which was scoured and bleached, was purchased from Tianjin Tianyi Dyeing limited company in North China.

#### 2.1.3. Chemical and reagents

Glycine was the biological reagent, which was of scientific research special grade, with MW of 75.07. Sodium periodate, glycerine and gelatin were of analytical reagent grade.

### 2.2. Dyeing with reaction solution between tea polyphenol and glycine

#### 2.2.1. Cotton modification

Cotton fabrics were placed in 3 g l^−1^ sodium periodate solution, and were treated for 1 h in a 50°C thermostat water bath. The treated cotton fabrics were placed in 0.1 M glycerine solution for 10 min to wash off residual sodium periodate solution. Then the treated cotton fabrics for oxidation treatment were placed in 2 g l^−1^ gelatin solution with a liquor ratio of 1 : 50, and were processed for 1 h in a 50°C thermostat water bath. Finally, the treated cotton fabrics were taken out from the thermostat water bath, washed under running water and dried in a drying oven.

#### 2.2.2. Dyeing procedure

Adding 50 g l^−1^ tea polyphenols and 50 g l^−1^ glycine into dyeing tanks (with natural pH), the fabrics were placed into it with a liquor ratio of 1 : 50. The dyeing experiment was processed in infrared dyeing equipment (Data Color Corporation, USA). The dyeing temperature was 100°C and the soaking time was 60 min, which started from 30°C with a heating rate of 3°C min^−1^. At the end of the dyeing process, the dyed fabrics were washed under running water, which was also carried out using 2 g l^−1^ neutral soap flakes at 100°C for 10 min to wash away residual uncombined pigment with fabrics. After soaping, fabrics were washed twice with water at 100°C and then under running water, followed by drying in a drying oven.

#### 2.2.3. Effect of filtration on dyeing of protein fabrics

Protein fabrics (wool and silk) were placed in dyeing tanks that contained residual reaction solution, both natural TP and natural TP+ glycine, respectively, with a liquor ratio of 1 : 50. The following dyeing process was similar to that in §2.2.2.

### 2.3. Measurements

#### 2.3.1. FTIR analysis

The FTIR spectrometer (Nicolet iS50, Thermo Fisher, USA) was employed to analyse structural changes both in the reaction solution and the dyed fabric. The infrared spectrums of the reaction solution and the wool fabrics (dyed and undyed) were obtained by employing the KBr pellet method.

#### 2.3.2. Particle size analysis

The volume distribution of the reaction solution particle diameter of dyed fabrics by employing natural TP was measured by a laser particle diameter analytical instrument (Beckman Coulter Inc, USA). The mean particle diameter of the reaction solution was measured.

#### 2.3.3. Colour characteristics

The CIE L*, a*, b*, C*, h and *Integ* values were measured by employing a Data colour 600 spectrophotometer (Data Color Corporation, USA) under photosource D65, with a 10° visual angle. The measurement result was the mean values from four different locations. The *Integ* value could be calculated according to the following equation:

2.1 Integ= F(X) + F(Y) + F(Z) , 

where F(X), F(Y) and F(Z) are pseudo-tristimulus values.

#### 2.3.4. Colour fastness

The rubbing, soaping and light fastness of dyed fabrics were measured on the basis of ISO 105-C01, ISO 105-X12 and ISO 105-B02, respectively. The rubbing fastness was tested by a rubbing fastness instrument (Y571B, China); the theory was to rub dry and wet cotton fabrics with dyed fabric, and then to evaluate the staining degrees of the rubbing cloth. The soaping fastness was measured by a soaping fastness instrument machine (SW-12, China). The method was to sew standard adjacent fabrics, which were placed in soap solution to agitate within the required time and temperature. The light fastness was tested by a light fastness instrument (Xenotest150 S +, ATLAS Corporation, USA); the theory was to illuminate dyed fabrics and a wool standard sample, and to contrast the colour change between the two sides and evaluate light fastness.

## 3. Results and discussion

### 3.1. Dyeing of cotton fabric with reaction solution between tea polyphenol and glycine

**Figure 2.**
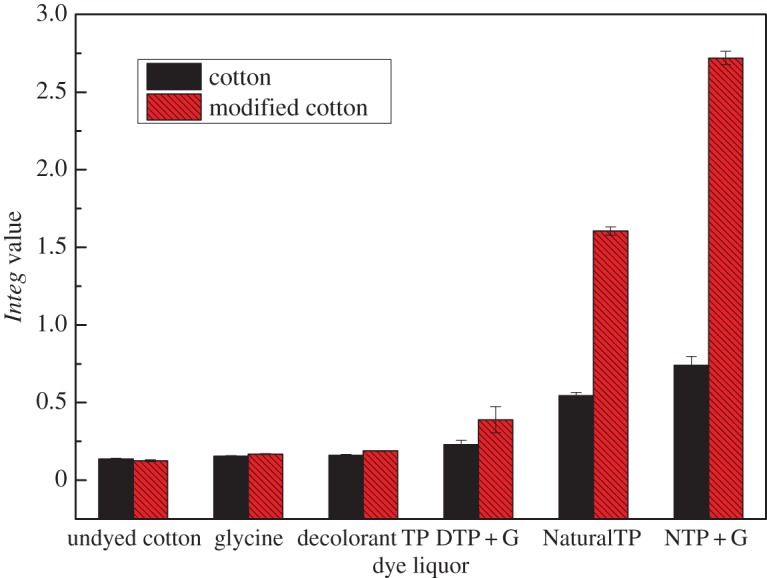
Effect of dye liquor on *Integ* values of dyed cotton fabric.

**Figure 3.**
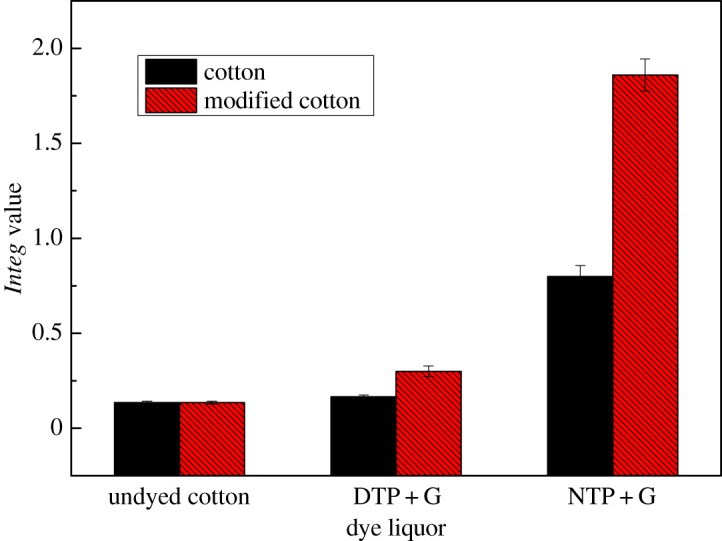
Effect of residual liquor on *Integ* values of dyed cotton fabric.

It was declared that the *Integ* values of modified cotton fabrics were greater compared to those of counterparts when dyed by identical dye liquor. The combination mode between the two sides was an ionic bond, on the grounds that cationic modification of cotton fabrics has been carried out. From figure 2, we could make out that the *Integ* values of dyeing liquor with natural tea polyphenols were bigger compared to that of colourless TP as a whole, the possible reason being that the structure of colourless TP was destroyed during the decolourization process. Additionally, we could discover that the *Integ* values of modified cotton fabrics were greater than those of counterparts when dyed by residual liquor (figure 3), on the grounds that the concentration of tea pigments of residual liquor was higher compared to the first reaction solution. On another level, the bonding forms were intermolecular hydrogen bonds as well as van der Waals' forces between tea pigments and the cotton fabric, and the combination mode was an ionic bond between tea pigments and the modified cotton fabric. In conclusion, glycine contributed to the formation of tea pigments owing to modification of the cotton fabrics by amination, and the colour strength of dyed modified cotton fabric was enhanced apparently compared to dyed cotton fabrics.

### 3.2. Dyeing of protein fabric with reaction solution between tea polyphenol and glycine

#### 3.2.1. Colour parameters of dyed protein fabrics

**Figure 4.**
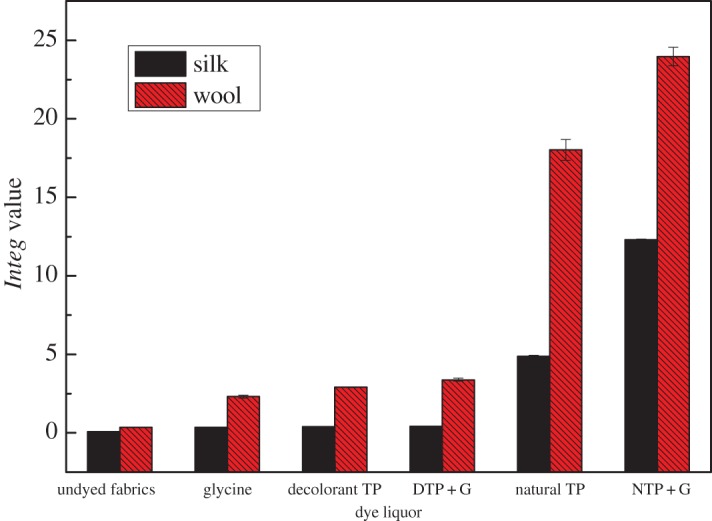
Effect of dye liquor on *Integ* values of dyed protein fabric.

It was explained that the *Integ* values of dyed wool fabrics were much greater compared to those of silk fabrics, and the *Integ* values of dye bath with natural TP+ glycine were also greater (figure 4). Furthermore, colour parameters a* and b* were positive values, and the hue angles of dyed fabrics were less than 90° according to tables 1 and 2. It could be demonstrated that dyed fabrics possessed red as well as yellow chroma brightness. On another level, the colour fastness of dyed protein fabrics was admirable according to tables 3 and 4, including rubbing fastness, washing fastness as well as light fastness. In addition, the average particle size of dyed residual liquor with natural TP was 717.0 nm according to figure 7; the structure of the silk fabric was relatively tight, and hence the residual liquor could not diffuse into the silk fabric.

**Table 1.** The colour parameters of dyed silk fabrics.

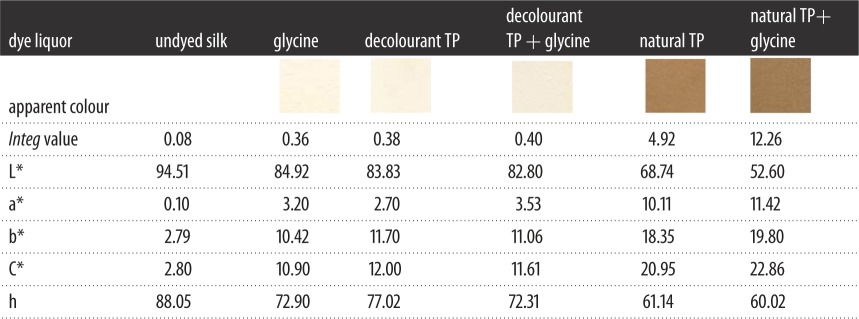

**Table 2.** The colour parameters of dyed wool fabrics.

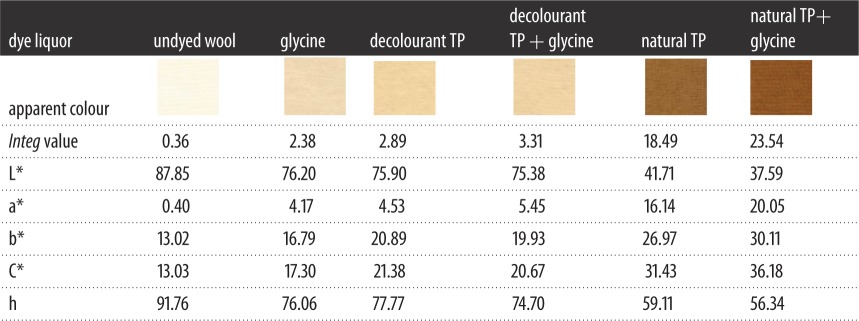

**Table 3:** The colour fastness of dyed silk fabrics. (Colour change (CC), staining on cotton fabric (SC), staining on wool fabric (SW), decolourant tea polyphenols + glycine (DTP + G), natural tea polyphenols + glycine (NTP + G).)

	rubbing fastness		washing fastness			
dye liquor	dry	wet	CC	SC	SW	light fastness
glycine	5	4–5	5	5	5	5
decolourant TP	5	4–5	5	5	5	4–5
DTP + G	4–5	4	4–5	4	4–5	4–5
natural TP	5	4–5	5	5	5	4–5
NTP + G	4–5	4	4–5	4	4–5	4–5

**Table 4:** The colour fastness of dyed wool fabrics. (Colour change (CC), staining on cotton fabric (SC), staining on wool fabric (SW), decolourant tea polyphenols (DTP), glycine (G).)

	rubbing fastness		washing fastness			
dye liquor	dry	wet	CC	SC	SW	light fastness
glycine	5	4–5	5	5	5	4–5
decolourant TP	5	4–5	5	5	5	4–5
DTP + G	4	4	4–5	4	4–5	4
natural TP	4–5	4–5	5	5	5	4–5
natural TP + G	4	4	4–5	4	4–5	4

**Figure 5.**
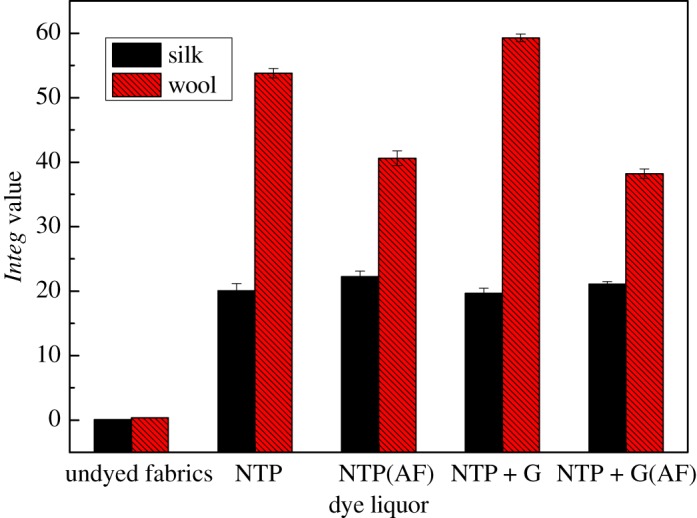
Effect of residual liquor on *Integ* values of dyed protein fabric.

It was declared that the *Integ* values of dyed wool fabrics were much greater compared to those of silk fabrics by employing residual liquor according to tables 5 and 6. Moreover, all the colour parameters a* and b* were positive values, and the hue angles of the dyed fabrics were less than 90°. It could be demonstrated that dyed fabrics possessed red as well as yellow chroma brightness because tea polyphenols have been transformed into tea pigments. The *Integ* values of dyed wool fabrics with natural tea polyphenols of residual liquor were bigger than those post-filtration according to figure 5, which made it clear that natural tea polyphenol particles could penetrate into wool fabric on the grounds that the structure of wool fabric was relatively loose and its scale layers would unfold under a temperature of 100°C. On another level, the colour fastness of dyed protein fabric with residual liquor was admirable according to tables 7 and 8, and could meet the application requirements.

**Table 5.** The colour parameters of dyed silk fabrics via using residual liquor.

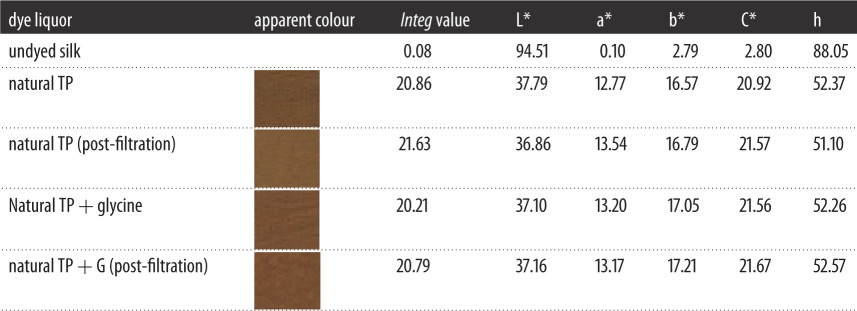

**Table 6.** The colour parameters of dyed wool fabrics via using residual liquor.

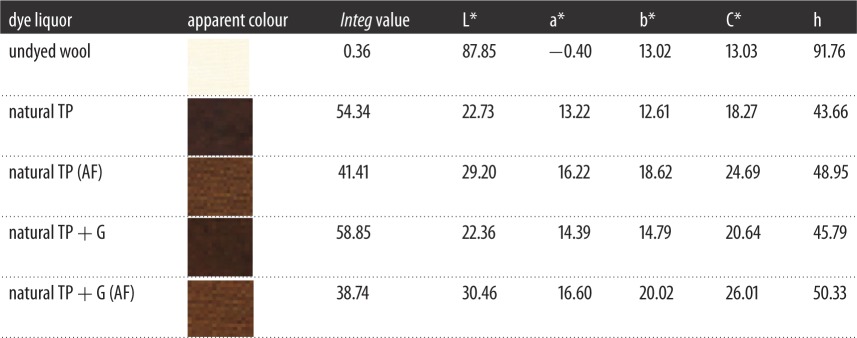

**Table 7:** The colour fastness of dyed silk fabrics via using residual liquor. Modified (M), unmodified (UM), tea polyphenols (TP).

	rubbing fastness		washing fastness			
dye liquor	dry	wet	CC	SC	SW	light fastness
decolourant TP + glycine (UM)	4–5	4	4–5	5	4	4–5
decolourant TP + glycine (M)	4–5	4	4–5	4	4–5	4–5
natural TP+ glycine (UM)	4–5	4	4–5	5	4	4–5
natural TP+ glycine (M)	4–5	4	4–5	4	4–5	4–5

**Table 8:** The colour fastness of dyed wool fabrics via using residual liquor. (Colour change (CC), staining on cotton fabric (SC), staining on wool fabric (SW), natural tea polyphenols + glycine (NTP + G), after filtration (AF).)

	rubbing fastness		washing fastness			
dye liquor	dry	wet	CC	SC	SW	light fastness
natural TP	4	4	4–5	4	4	4–5
natural TP (AF)	4–5	4	4–5	4–5	4–5	4–5
natural TP + G	4	4	4–5	4	4	4–5
natural TP + G (AF)	4–5	4	4–5	4–5	4–5	4–5

#### 3.2.2 FTIR analysis of dyed wool fabric and reaction solution

**Figure 6.**
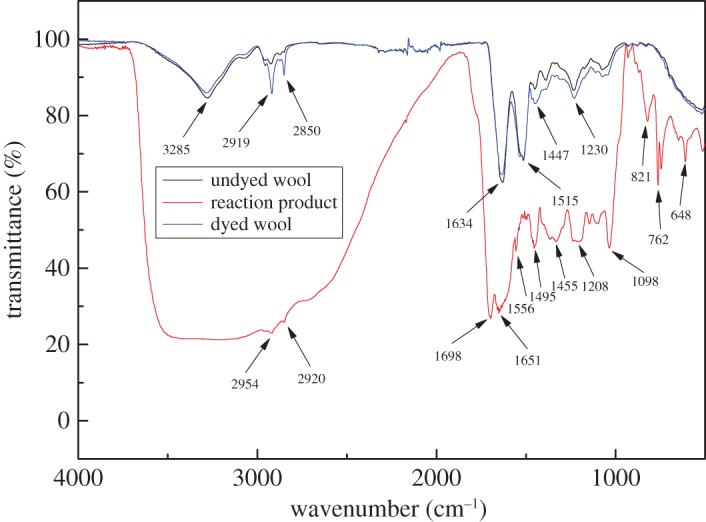
FTIR spectrums of undyed wool fabric, dyed wool fabric and reaction product between natural TP and glycine.

To demonstrate the structural characteristics of dyed wool fabric and reaction solution between natural TP and glycine, corresponding FTIR spectra were measured and are shown in figure 6. The peaks of hydroxyl groups could be made out for both undyed and dyed wool fabrics at 3285 cm^−1^. However, the characteristic peak of the hydroxyl group in reaction solution could not be found out, which illustrated that natural TP has been oxidized into polymers (tea pigments) under the conditions of high temperature as well as oxygen. The characteristic peaks of both undyed and dyed wool fabrics corresponding to N–H of amide stretch at 3285 cm^−1^; a C–H stretch range of 2800–3000 cm^−1^ was identified; which were at 2919 and 2850 cm^−1^ for dyed wool as well as 2954 cm^−1^ and 2920 cm^−1^ for reaction product between natural TP and glycine, respectively. The characteristic peaks of reaction product corresponding to C=O of carboxylic ester was at 1698 cm^−1^, in-plane C=O bending at 1651 cm^−1^, −NO_2_ stretching vibration at 1556 cm^−1^ and aromatic C=C stretching vibration at 1495 cm^−1^ as well as alkane C–H bending vibration at 1455 cm^−1^. It could be explained that natural TP has been oxidized into thearubigins and glycine has participated in the formation of tea pigments under the non-enzymatic browning reaction at high temperature. The characteristic peaks of reaction product correspond to alkane C–H skeletal vibration at 1208 cm^−1^, ether C–O–C stretching vibration at 1098 cm^−1^, aromatic C–H bending vibration at 762 cm^−1^ and alkyne C–H bending vibration at 648 cm^−1^. This makes clear that thearubigins have been transformed into theabrownin, and monomers connect to each other in the ether linkage.

#### 3.2.3. Particle size distribution of tea pigments via non-enzymatic oxidation reaction of natural tea polyphenol

**Figure 7.**
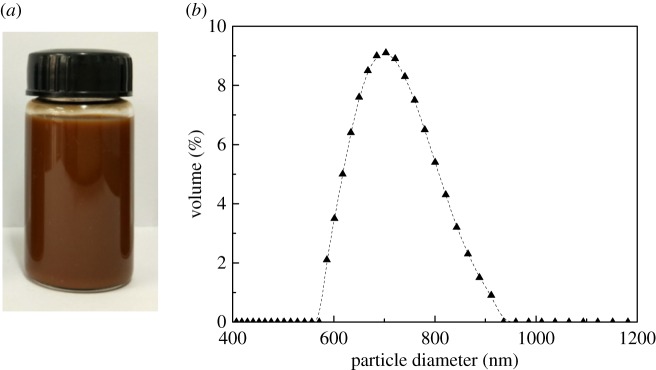
(*a*) Suspension of natural TP reaction solution; (*b*) size distribution of natural TP reaction solution.

Particle size measurement was performed to characterize the suspension system of natural TP non-enzymatic oxidation reaction solution. Figure 7 reveals the suspension and its size distribution. The Dv (10%), Dv (50%) and Dv (90%) of natural TP reaction solution were 615.5, 700.7 and 811.2 nm, respectively, and the mean grain size was 717.0 nm. The polydispersity index was −0.034, which indicated that particle size distribution of the suspension system was of relative centralization, and the levelling performance of dyed fabrics was excellent.

### 3.3. Reaction mechanism analysis

**Figure 8.**
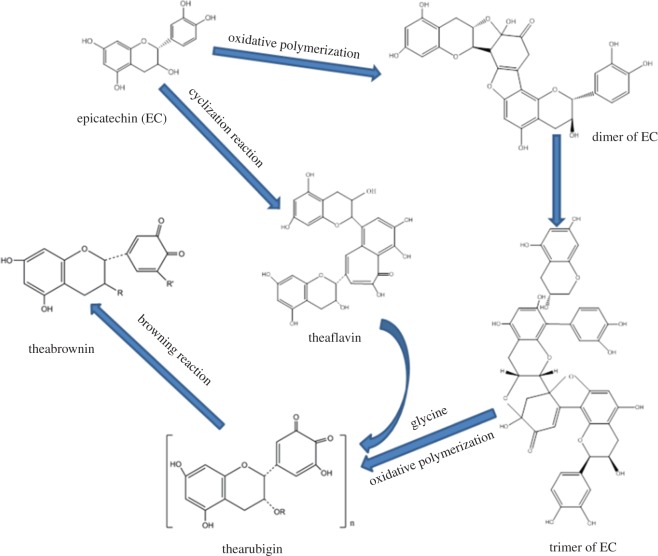
Major conversion pathways of non-enzymatic oxidation reaction between catechin and tea pigments.

Catechin is a key component of tea polyphenol, which accounts for 60%–80% of the total tea polyphenol content. The previous investigation of our team has demonstrated that catechins were apt for oxidative polymerization to generate dimers as well as trimers under alkaline conditions (figure 8). In addition, catechins were firstly inclined to proceed in cyclization reaction to form theaflavin and even dimers of theaflavin, which would be transformed into thearubigin and the ultimate oxidative product theabrownin via oxidative polymerization under alkaline or high-temperature conditions [19], and the concentration of tea pigments would further be enhanced through carrying out a non-enzymatic browning reaction when adding glycine into dyeing liquor. The quantity of phenolic hydroxyl groups became reduced with the progress of the conversion process. Accordingly, the antibacterial as well as anti-UV properties would be weakened gradually.

### 3.4. Dyeing mechanism analysis

The dyeing mechanism between tea pigments and cotton fabric was similar to that of vat dyestuffs; the adsorption mechanism accorded with the adsorption isotherm of the Freundlich model. Micromolecular tea polyphenols would be first absorbed onto the surface and then diffuse into the fabrics, which would gradually be transformed into macromolecular tea pigments and become fixated in the fabric via an *in situ* polymerization method with the constant rise in temperature. The diffusion model between tea pigments and cotton fabric conformed to the pore model of diffusion on the grounds that cotton fabric was a hydrophilic fibre. The bonding form was intermolecular hydrogen bonds and van der Waals’ force between tea pigments and cotton fabric, and the combination mode was ionic bonds between tea pigments and modified cotton fabric. As the bond energy of ionic bonds was much higher than that of intermolecular forces, the *Integ* values of dyed modified cotton fabric were greater than those of counterparts when dyed by uniform dyeing liquor.

The adsorption mechanism between tea pigments and protein fabrics was the adsorption isotherm of the Langmuir model [20], and the diffusion model accorded with the pore model on the grounds that both silk and wool fabrics were hydrophilic. The combination mode between tea pigments and protein fabrics were intermolecular forces as well as electrostatic force, but the *Integ* values of wool fabrics were greater than those of silk fabrics. The possible reason was that the content of amino acid was almost equal to that of carboxyl in wool fibre, while the quantity of amino acid was less than that of carboxyl in silk fibre.

## 4. Conclusion

In this investigation, a novel dyeing method was carried out, and the process involved greener and cleaner production on the grounds that toxic mordant or chemical materials were not added. Additionally, both tea polyphenol and amino acid were derived from renewable biomass resources, which was one of sustainable cleaner production technologies. Experimental results demonstrated that glycine contributed to the formation of tea pigments.

The *Integ* values of dyed protein fabrics by using residual liquor were much bigger than those of counterparts by employing the first reaction solution, and the *Integ* values of dyed wool fabrics were the greatest among all dyed fabrics when dyed by identical dye liquor. The investigation could be conducted many times because the concentration of the dyed residual liquor was relatively high, with the possibility of recycled dyeing.

## Data accessibility

This article has no additional data.
